# Transmission of Antimicrobial Resistant Bacteria at the Hajj: A Scoping Review

**DOI:** 10.3390/ijerph192114134

**Published:** 2022-10-29

**Authors:** Sara Alreeme, Hamid Bokhary, Adam T. Craig

**Affiliations:** 1 School of Population Health, Faculty of Medicine and Health, The University of New South Wales, Sydney 1466, Australia; 2 University Medical Center, Umm Al-Qura University, Makkah 24243, Saudi Arabia

**Keywords:** Hajj, antimicrobial resistance, systematic review

## Abstract

Background: The Hajj is an annual religious mass gathering event held in Makkah, Saudi Arabia. With millions of participants from across the globe attending the Hajj, the risk of importation, transmission, and global spread of infectious diseases is high. The emergence of antimicrobial resistant (AMR) bacteria is of worldwide concern and the Hajj poses a serious risk to its dissemination. This review aims to synthesize published literature on AMR bacteria acquisition and transmission associated with the Hajj. Methods: We searched electronic databases to identify literature published between January 1990 and December 2021. The search strategy included medical subject headings and keyword terms related to AMR bacteria and the Hajj. Results: After screening 2214 search results, 51 studies were included in the analysis. The review found 6455 AMR bacteria transmissions related to the Hajj. Thirty predominantly enteric or respiratory disease-causing AMR bacterial species were reported with isolates identified in cases on five continents. Most were male, aged above 50 years and were diagnosed in Makkah. Most cases were identified through hospital-based research; few cases were detected in community or primary health care settings. Conclusions: This review provides a contemporary account of knowledge related to AMR transmission at the Hajj. It emphasizes the need for the enhancement of surveillance for AMR bacteria globally.

## 1. Introduction

Antimicrobial resistance has emerged as a global threat to public health and health systems [1]. Antimicrobials are chemical compounds or drugs, including antibiotics, used therapeutically to stop the replication of disease-causing microorganisms in humans, animals, and plants [2]. Antimicrobial resistance (AMR) refers to a situation when microorganisms that would otherwise be susceptible to the antimicrobial agents undergo intrinsic changes over time that result in them developing an ability to survive (or resist) the effect of these agents [2]. AMR is accelerated by the over- and inappropriate use of antibiotics in agriculture and by humans [3].

Therapeutically, AMR has reduced the ability to effectively treat some disease-causing microorganisms, leading to increased mortality and morbidity [2]. This is perhaps most notably the case for *Mycobacterium tuberculosis* (TB), where around 29% of deaths among TB patients have been attributable to AMR TB [4]. Globally, the burden of disease associated with AMR in 2019 was estimated at 48 million disability-adjusted life years lost (DALYs), including 1.3-million deaths [5]. By 2050, AMR is predicted to be responsible for 10 million deaths annually and to cost the world’s economy US$100 trillion [6].

The epidemiology of AMR is complex. Globally, there are spatial differences in AMR bacteria occurrence (or at least detection) [7]. For example, in 2019, the global detected rate for methicillin-resistant *Staphylococcus aureus* (MRSA) was 12.1%; however, as claimed by the WHO, the rate is not representative as not all member countries provided their national rate [2]. This lack of reporting of AMR incidence and rates by countries may contribute to global monitoring and detection differences. Reasons for such limitations may include lack of reliable testing, availability of materials, and national AMR surveillance programs, especially in low-income countries [8]. The ability to move people and animals vast distances quickly through air and sea transport and the subsequent intermingling of people from high and low incidence regions is considered a major challenge to global efforts to mitigate the risks posed by AMR [9,10]. Major mass gathering events, such as the Hajj, exacerbate this risk [11].

Mass gatherings are defined by WHO as “the concentration of people at a specific location for a specific purpose over a set period and which has the potential to strain the planning and response resources of the country or community.” [12]. The Hajj pilgrimage is an annual 5–6-day mass gathering that attracts over 2 million people from across the world [13]. The Hajj involves a series of rituals in and around the holy city of Makkah, Saudi Arabia [13].

There have been public health challenges associated with the Hajj in the past. Notably, infectious disease events related to the Hajj have included a cholera epidemic in 1821 that resulted in an estimated 20,000 deaths [14] and meningitis outbreaks in 1987 and 2000, which led to mandating meningococcal vaccination for all pilgrims [15,16,17]. These events highlight the risk of transmission of infectious diseases at the event [18,19]. The threat of AMR bacteria transmission and global spread associated with the Hajj has been noted as a global health concern [20].

Several researchers have explored AMR-related outbreaks associated with the Hajj [21,22], and, in 2017, a systematic review on AMR events at the Hajj in the preceding 15 years (i.e., 2002–2017) was published [19]. Our research supplements this work and adds to the body of understanding by providing a contemporary (up to December 2021) account of the literature related to AMR and the Hajj. In addition, we offer an account of AMR pathogen-specific incidence and associated antibiotic resistance profiles.

## 2. Materials and Methods

### 2.1. Article Identification

A systematic search of the literature using four databases (CINAHL, Embase, PubMed, and Scopus) was conducted between 9 and 20 February 2022. The search was performed by SA. The search strategy involved Medical Subject Headings and keywords related to two domains. These were (i) AMR and (ii) the Hajj. The search strategy is presented in full in Supplementary Material S1.

### 2.2. Article Screening

The title and abstracts of identified articles were screened independently by two researchers (SA and HB) using established inclusion and exclusion criteria. To be included, an article needed to: be a research paper or lesson from the field-type articles; have been published between 1 January 1990 and 31 December 2021; be available in English; and include content related to AMR bacteria transmission, cases, or outbreaks associated with the Hajj pilgrimage. Articles that were systematic reviews, opinion pieces, or editorials were excluded, as were articles that studied organisms other than bacteria, focused on the biology of AMR, or were not available in English.

### 2.3. Data Extraction

Articles retained after screening were read in their entirety and data was extracted from them by SA and HB using a pre-developed data collection tool developed in Microsoft Excel (Microsoft Excel 365, Version 16.60, Microsoft Corporation, Redmond, Washington, DC, USA.) (Supplementary Material S2). Data extracted related to (i) the articles’ metadata (authors, title, date of publication, journal); (ii) the pathogen (bacterial species and genus, resistance profile, detection methods, clinical settings, and the number of isolates); and (iii) relationship with the Hajj pilgrimage (Supplementary Material S2).

### 2.4. Analysis

A mixed method approach to analysis was used. First, we conducted a meta-analysis extracting and tabulating data about AMR cases. Second, we used descriptive statistical method to summarize date. Proportions and confidence (CI) were calculated as per published statistical equations and methods [23,24,25]. Third, we used deductive and inductive thematic analysis methods to categorize information and distil pertinent themes. The study was undertaken in line with the preferred reporting items for systematic reviews and meta-analyses (PRISMA) guidelines [26]. Find the PRISMA checklist in Supplementary Material S3.

### 2.5. Ethics

Ethical approval was not required as this research relied on publicly available data.

## 3. Results

**Figure 1:**
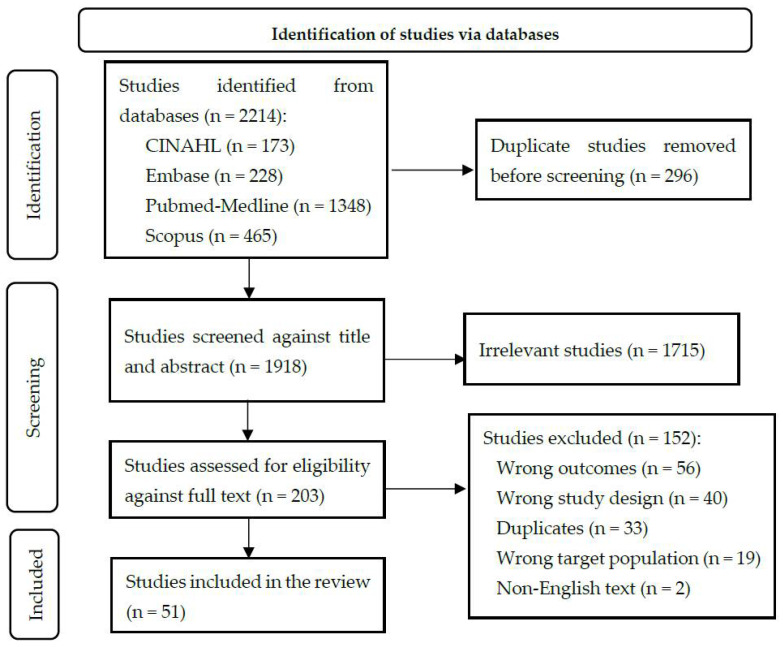
PRISMA flow diagram for the systematic scoping review that includes searches of the databases.

Of the 2214 studies identified during our systematic search, 51 were retained after screening. Figure 1 summarizes the data collection and screening process. The 51 articles kept were published between 1990 and 2020. The pooled population sample from these studies included in this review was 20,947 participants. The total number of female and male participants were 5566 and 8048, respectively (1:1.4; female to male ratio); sex was not reported by the research papers’ authors for 7333 (35.01%) participants. The average age of cases was 50 years (range from 14 to 101); this did not include one study that focused on children involved in a meningitis outbreak investigation [24]. A summary of these articles is presented in Table 1.

Twenty-six of the 51 papers reported the results of AMR-related research conducted in hospital settings [27,28,29,30,31,32,33,34,35,36,37,38,39,40,41,42,43,44,45,46,47,48,49,50,51,52] while eight were case reports [31,38,40,41,53,54,55,56]. One was an outbreak report [21], one hospital-based case-control study [45], and one article was an environmental to detect AMR in Makkah [57].

### 3.1. What AMR Bacteria Are Reported in the Literature?

Thirty unique AMR bacterial species from 6455 isolates were reported across the 51 articles. These were predominantly enteric or respiratory disease-causing bacteria.

#### 3.1.1. Enteric Disease-Causing AMR Bacteria

Among the enteric disease-causing AMR bacteria, *Escherichia coli* was the most reported in 15 studies [27,32,41,42,43,46,47,50,58,59,60,61,62,63,64]. *Klebsiella* spp. were reported in 13 studies [27,32,40,42,47,49,50,58,59,61,63,64,65], while *Acinetobacter* spp. [27,32,39,42,45,50,60,63,64] and *Pseudomonas* spp. [27,32,35,42,43,44,50,51,63] were both each reported in nine studies. Both *Enterobacter* spp. [27,32,42,47,50,64] and *Proteus* spp. [27,32,42,47,50,55] were reported in six studies, *Enterococcus* spp. in four studies [33,34,42,43], *Salmonella* spp. in three studies [27,62,66], and *Citrobacter* spp. in two studies [27,61]. Each of *Bacillus* spp. [57], *Bacteroides* spp. [27], *Brachybacterium* spp. [57], *Burkholderia* spp. [27], *Serratia* spp. [27], *Shigella* spp. [62], *Vibrio cholerae* [53], and *Yersinia enterocolitica* [62] were reported in single studies.

#### 3.1.2. Respiratory Disease-Causing AMR Bacteria

Among the respiratory disease-causing AMR bacteria: *Staphylococcus* spp. in 17 studies [28,30,33,36,37,42,43,48,52,55,57,61,63,64,67,68,69], *Streptococcus* spp. in seven study [33,42,65,70,71,72,73], *Neisseria meningitidis* in three studies [21,22,54], *Haemophilus influenzae* was reported in two studies [27,32], *Mycobacterium tuberculosis* in two studies [74,75], and *Stenotrophomonas maltophilia* in one study [38].

#### 3.1.3. Other AMR Bacteria

Six non-enteric/non-respiratory disease-causing AMR bacteria were identified. These were *Brucella* spp. [27], *Ewingella Americana* [31], *Helicobacter pylori* [29], *Microbacterium* spp. [57], *Micrococcus* spp. [57], and *Shewanella xiamenensis* [56].

All the organisms reported were found to have resistance to one or more antibiotic class. These results are presented in Table 2. In summary, resistance to beta-lactams class antibiotics was reported for 4856 AMR bacterial isolates in 45 studies [22,27,28,29,30,31,32,33,35,36,37,38,39,40,41,42,43,44,45,46,47,48,49,50,51,52,53,55,56,57,58,59,60,61,62,63,64,65,66,67,68,69,70,71,72], to quinolones (including fluoroquinolones) for 1611 AMR bacteria in 24 studies [22,27,29,32,33,35,38,40,42,43,44,45,46,49,50,55,59,60,61,63,64,70,71,72], to macrolides for 1161 AMR bacteria in 17 studies [27,28,29,33,37,43,55,57,61,64,67,68,69,70,72,73], to aminoglycosides for 2893 AMR bacteria in 27 studies [27,28,31,32,33,35,37,38,40,42,43,44,45,50,51,57,58,59,60,61,63,64,66,67,68,74,75], and to sulphonamides for 2504 AMR bacteria in 21 studies [21,27,28,31,32,33,35,38,40,43,45,53,57,59,60,61,64,67,70,71,73].

**Table 1:** Transmission of antimicrobial resistant bacteria in the Hajj.

Author [Ref.]	Year	AMR Bacteria	Antibiotic Resistance	Study Type	Summary
Salih, M. A., et al. [21]	1990	*Neisseria meningitidis*	Sulfadiazine	Outbreak investigation	The outbreak investigation reported 45 AMR-positive Sudanese pilgrims recently returned to Sudan from the Hajj.
Ng, P. P. and Taha, M. [53]	1994	*Vibrio cholerae*	Tetracycline, ampicillin, chloramphenicol, and trimethoprim/sulfamethoxazole	Case report	The case study reported that tetracycline-resistant *Vibrio cholera* was found in three pilgrims who recently returned to Malaysia from the Hajj.
Yousuf, M. and Nadeem, A. [54]	1995	*Neisseria meningitidis*	Cloxacillin	Case report	the case study reported that cloxacillin-resistant *Neisseria meningitidis* was found in two American and Indonesian male pilgrims admitted to a hospital in Madinah, Saudi Arabia.
Fatani, M. I., et al. [67]	2002	*Staphylococcus aureus*	Penicillin, erythromycin, cephalothin, trimethoprim/sulfamethoxazole, clindamycin, tetracycline, gentamicin, and oxacillin	Cross-sectional study (prospective)	The study reported 47 MRSA isolated from pyodermas patients admitted to a hospital in Makkah during the Hajj season.
Asghar, A. H. [27]	2006	*Escherichia coli, Pseudomonas* spp., *Acinetobacter* spp., *Klebsiella* spp., *Serratia* spp., *Enterobacter* spp., *Proteus* spp., *Salmonella* spp., *H. influenzae, Citrobacter* spp., *Bacteroides* spp., *Burkholderia* spp., *Brucella* spp.	Ampicillin, cefepime, cephalothin, ceftazidime, amoxicillin/clavulanic acid, cefoxitin, piperacillin/tazobactam, piperacillin, gentamicin, imipenem, aztreonam, amikacin, ciprofloxacin, trimethoprim/sulfamethoxazole, penicillin, oxacillin, erythromycin, and clindamycin	Cross-sectional study (prospective)	The study reported 1530 AMR cases (septicaemic patients) from hospitals in Makkah.
Asghar, A. H. and Momenah, A. M. [28]	2006	*Staphylococcus aureus*	Methicillin, penicillin, ampicillin, oxacillin, erythromycin, cephalothin, gentamicin, Oxytetracycline, and trimethoprim/sulfamethoxazole	Cross-sectional study (prospective)	The study reported 199 MRSA cases from hospitals in Makkah, of which 157 were found to have MDR.
Karima, T. M., et al. [29]	2006	*Helicobacter pylori*	Metronidazole, erythromycin, amoxicillin, tetracycline, and ciprofloxacin	Cross-sectional study (prospective)	The study reported that *H. pylori* was found in 18 patients (from a general hospital in Makkah) with resistance to at least one antibiotic.
Memish, Z. A., et al. [30]	2006	*Staphylococcus aureus*	Methicillin	Cross-sectional study (prospective)	The study reported six MRSA isolates from pilgrims during the Hajj of 2004.
Bukhari, S. Z., et al. [31]	2008	*Ewingella americana*	Amikacin, amoxicillin/clavulanic acid, ampicillin/sulbactam, ampicillin, cefazolin, cefepime, cefotaxime, ceftazidime, ceftriaxone, cefuroxime, cephalothin, gentamycin, imipenem, piperacillin/tazobactam, piperacillin, tetracycline, ticarcillin/clavulanic acid, tobramycin, trimethoprim/sulfamethoxazole	Case report	The case report of an AMR *E. americana* strain, isolated from an Indonesian pilgrim admitted to a hospital in Makkah during the Hajj.
Asghar, A. H. and Faidah, H. S. [32]	2009	*Escherichia coli, Klebsiella. pneumoniae, Klebsiella* spp., *P. aeruginosa, A. baumannii, Proteus* spp., *H. influenzae, Enterobacter* spp.	Cephalothin, cefoxitin, cefuroxime, ceftazidime, cefotaxime, ceftriaxone, ampicillin, aztreonam, piperacillin, piperacillin/tazobactam, amoxicillin/clavulanic acid, imipenem, meropenem, imipenem/cilastatin, amikacin, gentamycin, tobramycin, ciprofloxacin, levofloxacin, norfloxacin, nalidixic acid, norfloxacin/ciprofloxacin, tetracycline, trimethoprim/sulfamethoxazole, and nitrofurantoin	Cross-sectional study (prospective)	The study found 1046 g-negative bacteria isolated from patients in Makkah hospitals were resistant to at least one antibiotic.
Abulreesh, H. H. and Organji, S. R. [69]	2011	*Staphylococci*	Erythromycin, colistin, penicillin, oxacillin, vancomycin	Cross-sectional study (prospective)	The study reported 19 vancomycin-resistance *Staphylococci*, isolated from food and food handlers in Makkah.
Asghar, A. H. [33]	2011	*Staphylococcus aureus, Streptococcus pyogenes, Streptococcus agalactiae, Streptococcus pneumoniae, Streptococcus viridans, Enterococcus Fecalis*, and *Enterococcus* spp.	Cephalexin, cefazolin, cefoxitin, cefotaxime, ceftriaxone, ceftazidime, cefuroxime, ceftizoxime, penicillin, ampicillin, ampicillin/sulbactam, oxacillin, aztreonam, amoxicillin/clavulanic acid, imipenem/cilastatin sodium, gentamicin, neomycin, amikacin, ciprofloxacin, ciprofloxacin/norfloxacin, nalidixic acid, gemifloxacin, levofloxacin, vancomycin, erythromycin, clindamycin, quinupristin/dalfopristin, linezolid, tetracycline, chloramphenicol, trimethoprim/sulfamethoxazole, rifampicin, nitrofurantoin, and polymyxin B	Cross-sectional study (prospective)	The study of patients admitted to hospitals in Makkah found that the most common resistance reported was against beta-lactams.
El-Amin, N. M. and Faidah, H. S. [34]	2011	*Enterococci*	Vancomycin	Cross-sectional study (retrospective)	The retrospective study reported vancomycin-resistant Enterococci infections in seven patients from hospitals in Makkah.
Asghar, A. H. [35]	2012	*Pseudomonas aeruginosa*	Amikacin, amoxicillin/clavulanic acid, ampicillin, aztreonam, cefepime, cefotaxime, gentamycin, ceftriaxone, cefoxitin, ceftazidime, cefuroxime, cephalothin, ciprofloxacin, imipenem, meropenem, piperacillin, piperacillin/tazobactam, tetracycline, and trimethoprim/sulfamethoxazole	Cross-sectional study (prospective)	The study reported that metallo-beta-lactamase (MBL) -producing *P. aeruginosa* were identified in 76 patients (from 30 nationalities) admitted to hospitals in Makkah.
Asghar, A. H. [36]	2014	*Staphylococcus aureus*	Methicillin	Cross-sectional study (prospective)	The study reported that MRSA was identified in 114 patients admitted to hospitals in Makkah, of which 100 carried the mecA gene.
Khan, M. M., et al. [37]	2014	*Staphylococcus epidermidis*, *Staphylococcus haemolyticus*, *Staphylococcus hominis-homin*, *Staphylococcus hominis-novo*, *Staphylococcus warneri*, *Staphylococcus hominis*, *Staphylococcus capitis*, *Staphylococcus lugdunensis*, and *Staphylococcus auricularis*	Ampicillin, amoxicillin/clavulanic acid, azithromycin, clindamycin, daptomycin, erythromycin, gentamicin, oxacillin, penicillin, and quinupristin/dalfopristin	Cross-sectional study (prospective)	The study reported that 189 coagulase negative staphylococci (CoNS) isolates (from neonates admitted to a hospital in Makkah) were resistant to at least one of the tested antibiotics.
Abdel-Haleem, A. M., et al. [38]	2015	*Stenotrophomonas maltophilia*	Beta-lactams, cephalosporins, carbapenems, aminoglycosides, fluoroquinolones, tetracyclines, polymyxin, trimethoprim, gentamicin, and tigecycline	Case report	The case study reported MDR *S. maltophilia* strain was isolated from a patient with reoccurring urinary tract infection admitted to a tertiary hospital in Makkah.
Alyamani, E. J., et al. [39]	2015	*Acinetobacter baumannii*	Cefepime, ceftazidime, and multidrug resistance	Cross-sectional study (prospective)	The study reported 100 MDR *A. baumannii* isolates collected from patients admitted to hospitals in Makkah.
Memish, Z. A., et al. [70]	2015	*Streptococcus pneumoniae*	Penicillin, amoxicillin, cefotaxime, chloramphenicol, clindamycin, erythromycin, levofloxacin, moxifloxacin, tetracycline, or sulfamethoxazole/trimethoprim	Cross-sectional study (prospective)	The study reported 137 AMR positive pilgrims returning from the Hajj to their home countries (Algeria, Chad, Comoros, Egypt, Ethiopia, Guinea, India, Indonesia, Libya, Mauritania, Nigeria, and Sudan).
Olaitan, A. O., et al. [66]	2015	*Salmonella enterica*	Colistin, amoxicillin, amoxicillin/clavulanic acid, ceftriaxone, aztreonam, ceftazidime, imipenem, and gentamicin	Cross-sectional study (prospective)	The study reported MDR *Salmonella* in five French pilgrims who recently returned to Marseille, France after the Hajj.
Algowaihi, R., et al. [40]	2016	*Klebsiella pneumoniae*	Amikacin, amoxicillin/clavulanic acid, ampicillin, cefazolin, cefepime, cefotaxime, cefoxitin, ceftazidime, cefuroxime, ciprofloxacin, ertapenem, fosfomycin, gentamicin, imipenem, levofloxacin, meropenem, mezlocillin, moxifloxacin, nitrofurantoin, norfloxacin, piperacillin/tazobactam, tetracycline, tigecycline, tobramycin, trimethoprim, and sulfamethoxazole	Case report	The study reported MDR *K. pneumoniae* strain isolated from a female patient with a urinary tract infection, admitted to a tertiary hospital in Makkah.
Alyamani, E. J., et al. [41]	2016	*Escherichia coli*	Penicillin, carbapenems, cephamycin, tetracycline, chloramphenicol, acriflavine, and polymyxin	Case report	The case study reported MDR uropathogenic *Escherichia coli* O25b:H4 strain isolated from a male patient admitted to a hospital in Makkah.
Haseeb, A., et al. [42]	2016	*Acinetobacter baumannii, Escherichia coli, Enterobacter cloacae, Enterococcus* spp., *Klebsiella pneumoniae, Pseudomonas aeruginosa, Proteus mirabilis, Salmonellae, Staphylococcus aureus, Staphylococcus epidermidis*, and *Streptococcus* spp.	Amoxicillin/clavulanic acid, ampicillin, aztreonam, cefazolin, cefepime, cefoxitin, cefuroxime, ceftazidime, cefotaxime, ceftriaxone, cephalothin, ertapenem, imipenem, meropenem, oxacillin, penicillin, piperacillin/tazobactam, ticarcillin, mezlocillin, amikacin, gentamicin, tobramycin, ciprofloxacin, levofloxacin, moxifloxacin, nalidixic acid, and norfloxacin	Cross-sectional study (retrospective)	The retrospective study reported 214 AMR bacterial isolates collected from pilgrims who visited emergency care departments of Makkah hospitals.
Johargy, A. K. [43]	2016	*Enterococcus faecium, Escherichia coli, Staphylococcu aureus* and *P. aeruginosa*	Amoxicillin/clavulanic acid, amikacin, ceftazidime, cephalothin, erythromycin, gentamycin, chloramphenicol, oxacillin, clindamycin, ciprofloxacin, penicillin, vancomycin, piperacillin, cefotaxime, nalidixic acid, nitrofurantoin, oxacillin, and sulfamethoxazole	Cross-sectional study (prospective)	The study reported 129 AMR bacterial isolates collected from diabetic patients from hospitals in Makkah.
Khan, M. A. and Faiz, A. [44]	2016	*Pseudomonas aeruginosa*	Amikacin, aztreonam, cefepime, ceftazidime, ciprofloxacin, gentamicin, imipenem, levofloxacin, meropenem, piperacillin, piperacillin/tazobactam, and ticarcillin	Cross-sectional study (prospective)	The study reported 27 AMR and 8 MDR *P. aeruginosa* isolates collected from patients admitted to hospitals in Makkah.
Leangapichart, T., et al. [58]	2016	*Escherichia coli and Klebsiella pneumoniae*	Ticarcillin/clavulanic acid, ceftriaxone, and gentamicin	Cross-sectional study (prospective)	The study reported 28 AMR *Escherichia coli* or *K. pneumoniae* isolates collected from French pilgrims before and after the Hajj.
Leangapichart, T., et al. [59]	2016	*Escherichia coli and Klebsiella pneumoniae*	Amoxicillin, amoxicillin/clavulanic acid, ceftriaxone, ciprofloxacin, fosfomycin, gentamicin, nalidixic acid, and sulfamethoxazole/trimethoprim	Cross-sectional study (prospective)	A letter to editor reporting the presence of colistin-resistance gene, mcr-1 among 23 French pilgrims (before and after the Hajj of 2013 and 2014), ten of which were *Escherichia. coli*, and one was *K. pneumoniae*.
Leangapichart, T., et al. [60]	2016	*Acinetobacter baumannii* and *Escherichia coli*	Aztreonam, cefoxitin, ceftriaxone, cefotaxime, amoxicillin/clavulanic acid, ticarcillin/clavulanic acid, amoxicillin, tobramycin, gentamicin, ciprofloxacin, ofloxacin, imipenem, and sulfamethoxazole/trimethoprim	Cross-sectional study (prospective)	A study reporting MDR *A. baumannii* and *Escherichia coli* among 43 French pilgrims (before and after returning from the Hajj of 2014)
Marglani, O. A., et al. [61]	2016	*Staphylococcus aureus, Klebsiella pneumoniae, Klebsiella oxytoca, Escherichia coli, Enterobacter* spp., and *Citrobacter* spp.	Amoxicillin/clavulanic acid, ampicillin, cefoxitin, cefepime, ceftazidime, ceftriaxone, ciprofloxacin, levofloxacin, gentamicin, imipenem, piperacillin/tazobactam, sulfamethoxazole/trimethoprim, clindamycin, azithromycin, erythromycin, tetracycline, and vancomycin	Cross-sectional study (prospective)	A study reporting 57 AMR isolates collected from pilgrims with acute rhinosinusitis) during the Hajj of 2014.
Memish, Z. A., et al. [71]	2016	*Streptococcus pneumoniae*	Erythromycin, clindamycin, tetracycline, penicillin, amoxicillin, cefotaxime, levofloxacin, moxifloxacin, chloramphenicol, and sulfamethoxazole/trimethoprim	Cross-sectional study (prospective)	The study reported 94 AMR *S. pneumoniae* isolates collected from pilgrims (before and during the Hajj of 2013) from 12 countries in Africa, Asia, USA, and Europe.
Abd El Ghany, M., et al. [62]	2017	*Salmonella* spp., *Shigella* spp., and *Escherichia coli*, *Yersinia enterocolitica*	Beta-lactams	Cross-sectional study (prospective)	The study reported 70 AMR bacterial isolates collected from pilgrims (from 40 different countries) who acquired enteric infections during the Hajj of 2011 to 2013.
Abulreesh, Hussein H., et al. [68]	2017	*Staphylococcus aureus*	Amoxicillin/clavulanic acid, ampicillin, azithromycin, cefoxitin, clindamycin, erythromycin, fusidic acid, gentamicin; imipenem, oxacillin, penicillin, and tetracycline	Cross-sectional study (prospective)	The study reported 50 AMR *S. aureus* isolates collected from clinical laboratories in Makkah.
Al-Gethamy, M. M., et al. [45]	2017	*Acinetobacter baumannii*	Ceftazidime, ciprofloxacin, imipenem, trimethoprim, amikacin, gentamicin.	Casecontrol study	The case–control study reported MDR *A. baumannii* isolates (collected from patients admitted to a hospital in Makkah), that mainly resistance to imipenem and gentamycin with 83% and 73%, respectively.
Alyamani, E. J., et al. [46]	2017	*Escherichia coli*	Ampicillin, cefoxitin, ciprofloxacin, cefepime, aztreonam, cefotaxime, and ceftazidime	Cross-sectional study (prospective)	The study reported 58 AMR *Escherichia coli* isolates, collected from pilgrims admitted hospitals in Makkah, during the Hajj of 2014 and 2015.
Ahmed Khan, T., et al. [55]	2018	*Staphylococcus aureus* and *Proteus* spp.	Ampicillin, ciprofloxacin, fusidic acid, penicillin, cefuroxime, ceftriaxone, cefixime, erythromycin, cefoxitin, and tetracycline	Case report	The case study reported MDR *S. aureus* and *proteus* spp. isolates, collected from a burn aggravated infected foot wart in a pilgrim who came from Pakistan to perform the Hajj of 2017.
Ganaie, F, et al. [72]	2018	*Streptococcus pneumoniae*	Penicillin, cefotaxime, levofloxacin, erythromycin, tetracycline, and sulfamethoxazole/trimethoprim	Cross-sectional study (prospective)	The study reported 145 AMR *S. pneumoniae* isolates, collected from Indian pilgrims before and after returning from the Hajj of 2016, with higher AMR rates within the post-Hajj samples.
Khan, M. A., et al. [47]	2019	*Klebsiella pneumoniae, Escherichia coli, E. cloacae* and *Proteus mirabilis*	Cephalosporins (ceftazidime, cefotaxime, ceftriaxone, cefepime) and carbapenems	Cross-sectional study (prospective)	The study reported 27 carbapenemase Enterobacteriaceae isolates (collected from patients admitted to hospitals in Makkah), of which 21 were carbapenemase producing *K. pneumoniae*.
Mater, M. E., et al. [48]	2020	*Staphylococcus aureus*	methicillin	Cross-sectional study (retrospective)	The retrospective study reported 92 MRSA isolates, collected from burn and paediatric patients admitted to a hospital in Makkah, from January 2016 to January 2017.
Sambas, MFMK, et al. [74]	2020	*Mycobacterium tuberculosis*	Streptomycin, isoniazid, ethambutol, and rifampicin,	Cross-sectional study (prospective)	The study reported 27 AMR Tuberculosis (TB) isolates and 8 MDR-TB isolates, collected from TB patients admitted to a hospital in Makkah.
Willerton, L., et al. [22]	2020	*Neisseria meningitidis*	Penicillin and ciprofloxacin	Cross-sectional study (retrospective)	The retrospective study reported penicillin and ciprofloxacin *N. meningitidis* isolates, from *N. meningitidis* patients returning from Makkah, after preforming umrah pilgrimage to England.
Ahmed, O. B., et al. [49]	2021	*Klebsiella pneumoniae*	Amoxicillin/clavulanic acid, ciprofloxacin, cefotaxime, ampicillin, aztreonam, cefuroxime, cefepime, and imipenem	Cross-sectional study (retrospective)	The retrospective study reported 51 aminoglycoside-resistant *K. pneumonia* isolates, collected from hospital admitted patients in Makkah.
Ahmed, Omar B., et al. [50]	2021	*Klebsiella pneumoniae, Escherichia coli, P. aeruginosa, A. baumannii, K. oxytoca, P. mirabilis*, and *Enterobacter* spp.	Tobramycin, kanamycin, gentamicin, neomycin, amikacin, streptomycin, cefotaxime, amoxicillin/clavulanic acid, and ciprofloxacin	Cross-sectional study (retrospective)	The retrospective study reported 69 g-negative bacterial isolates with aminoglycoside-resistant gene(s), collected from hospital admitted patients in Makkah.
Al-Hayani, A. M, et al. [75]	2021	*Mycobacterium tuberculosis*	isoniazid, streptomycin, ethambutol, rifampicin, and pyrazinamide	Cross-sectional study (retrospective)	The retrospective study reported 93 TB patients with AMR, where data collected from the registry of the Central TB Laboratory in Makkah.
Al-Zahrani, I. A. and Al-Ahmadi, B. M. [51]	2021	*Pseudomonas aeruginosa*	Beta-lactams, amikacin, and colistin	Cross-sectional study (prospective)	The study reported 35 carbapenem-resistant *P. aeruginosa* isolates, collected from 26 hospital admitted patients in Makkah.
Alghamdi, S. [63]	2021	*Acinetobacter species, Klebsiella pneumonia, Escherichia coli, Staph. aureus species and Pseudomonas aeruginosa.*	Cefoxitin, penicillin, gentamicin, ampicillin, methicillin, clindamycin, sulfamethoxazole/trimethoprim, vancomycin, Trimoxazole, linezolid, ciprofloxacin, levofloxacin, cefuroxime, amikacin, ceftazidime, cefepime, and cefoperazone/sulbactam	Cross-sectional study (retrospective)	The retrospective study reported 123 AMR bacterial isolates, 15 were MRSA, and 6 MDR *Acinetobacter* spp. isolates, collected from cancer patients who were admitted to Makkah hospitals.
Harimurti, K., et al. [73]	2021	*Streptococcus pneumoniae*	Erythromycin, clindamycin, chloramphenicol, penicillin, sulfamethoxazole/trimethoprim, and tetracycline	Cross-sectional study (prospective)	The study reported 85 AMR *S. pneumoniae* isolates, collected from Indonesian pilgrims before and after the Hajj of 2015.
Hoang, V. T., et al. [64]	2021	*Enterobacter aerogenes, Escherichia coli, Klebsiella pneumoniae, Enterobacter cloacae, Staphylococcus aureus, Acinetobacter baumannii*	Imipenem, doripenem, piperacillin/tazobactam, fosfomycin, sulfamethoxazole/trimethoprim, ciprofloxacin, ticarcillin, ticarcillin/clavulanic acid, tobramycin, fusidic acid, erythromycin, methicillin, amoxicillin/clavulanic acid, cefepime, ceftriaxone, and colistin	Cross-sectional study (prospective)	The study reported 81 MDR isolates, collected from pilgrims from Marseille, France, during the Hajj of 2017 and 2018, of which 23 were isolated from pre-Hajj, and 52 from post-Hajj.
Leangapichart, T., et al. [56]	2021	*Shewanella xiamenensis*	Amoxicillin, amoxicillin/clavulanic acid, and ticarcillin/clavulanic acid	Case report	The case study reported two beta-lactam resistant *S. xiamenensis* strains, isolated from a Moroccan pilgrim from France, one before and one during travels to the Hajj of 2013.
Mohd Baharin, I. E., et al. [65]	2021	*Streptococcus pneumoniae* and *Klebsiella pneumoniae*	Beta-lactams and macrolide	Cross-sectional study (prospective)	The study reported 14 AMR *K. pneumoniae and S. pneumoniae* isolates, collected from Malaysian pilgrims returning to Kelantan, Malaysia from the Hajj.
Turkstani, M. A., et al. [57]	2021	*Staphylococcus* spp., *Micrococcus* spp., *Bacillus* spp., *Microbacterium* spp., *Geobacillus* spp., *Brachybacterium* spp.	Penicillin, erythromycin, ampicillin, chloramphenicol, clindamycin, gentamicin, sulfamethoxazole/trimethoprim, fusidic acid, oxacillin, and cefepime	Environmental study	The environmental research study reported 40 AMR bacterial isolates, collected from surface swab samples from two membership-based gyms in Makkah.
Haseeb, A., et al. [52]	2022	*Staphylococcus aureus, non-fermenter Gram-negative bacilli, Enterobacteriaceae, Enterococci.*	Methicillin, beta-lactams, carbapenems, third generation cephalosporins, and vancomycin.	Cross-sectional study (prospective)	The study reported 106 AMR isolates and 46 MRSA isolates, collected from patients admitted to Makkah hospitals.

ESBL = extended spectrum beta-lactamase; MDR = multidrug resistant; NA = not available.

The next part of the manuscript discusses key data by key theme. The themes are (i) how AMR was detected; (ii) the geospatial distribution of AMR cases reported in the literature; and (iii) the resistance profile. For consistency with the previous section and where relevant, results are presented as those related to enteric disease-causing and respiratory-illness-causing AMR.

### 3.2. The Methods of Detecting AMR Bacteria

The method by which AMR was detected was reported in 46 of the 51 papers. The most common method used was disc diffusion (*n* = 26 studies) [21,27,28,29,30,34,36,43,47,49,50,54,55,56,57,58,60,61,64,66,67,68,69,70,71,73], followed by polymerized chain reaction (PRC) (*n* = 21 studies) [35,36,38,39,40,41,46,47,50,51,56,58,59,60,62,64,65,66,68,70,71]. Automated identification systems were used (*n* = 14 studies) [30,31,33,35,37,39,43,44,46,51,61,63,72,75], two of which used molecular assay kits also [30,46]. Gradient strip diffusion was used in two studies [22,68]. Data from patients’ medical records were extracted in five studies to determine AMR status [32,42,45,48,74]; the specific diagnostic methods used were not reported.

### 3.3. The Geospatial Distribution of AMR Cases Reported

Our review found evidence for AMR bacteria among pilgrims travelling to the Hajj or returning from it from five continents. Nine studies reported 1422 Hajj-associated AMR cases from 20 Asian countries [53,54,55,62,65,70,71,72,73]; four studies reported 286 cases from 16 African countries [21,62,70,71]; two studies reported six cases from two North American countries [62,71]; nine studies reported 2069 cases from two European countries [22,56,58,59,60,62,64,66,71]. One study reported one case from Australia [62]. Most research articles (*n* = 34) and AMR resistant isolates (5686 resistant isolates of 8035 tested samples (70.77% [95% CI: 69.77%–71.76%]) were identified in Makkah, the city in which the Hajj takes place and from where most pilgrims come [27,28,29,30,31,32,33,34,35,36,37,38,39,40,41,42,43,44,45,46,47,48,49,50,51,52,57,61,63,67,68,69,74,75].

#### 3.3.1. Geospatial Distribution of Enteric Disease-Causing AMR Bacteria

Eight articles discussed 2802 enteric disease-causing AMR bacteria isolates (out of 3315 tested) among travelling Hajj pilgrims, two studies for pilgrims returning to Asia (Malaysia) [53,65], five of pilgrims returning to Europe (France) [58,59,60,64,66]. One had a mixed cohort of pilgrims from 40 countries (Afghanistan, Algeria, Australia, Azerbaijan, Bangladesh, Benin, Burma, Canada, Chad, China, Egypt, Ethiopia, Ghana, Guinea, India, Indonesia, Iraq, Jordan, Kazakhstan, Mali, Malaysia, Morocco, Mauritania, Nepal, Niger, Nigeria, Oman, Pakistan, Palestine, Philippines, Saudi Arabia, Somalia, Sudan, Syria, Tunisia, Turkey, UK, Union des Comoros, USA, and Yemen) [62].

#### 3.3.2. Geospatial Distribution of Respiratory Illness-Causing AMR Bacteria

Eleven articles studied 1330 respiratory illness-causing AMR bacteria (out of 2145 tested) isolated from travelling pilgrims, and five studies for pilgrims who had travelled from Asia (India and Pakistan) to the Hajj [55,72], or vice versa (India, Indonesia, Saudi Arabia, and Malaysia) [54,65,72,73]. Moreover, one study looked at an outbreak caused by returning pilgrims to Africa (Sudan) [21]. Three studies reported AMR among pilgrims returning to Europe (the UK and France) [22,56,64], or vice versa (France) [56,64]. Two studies looked at respiratory illness-causing AMR bacteria among pilgrims traveling from different countries (12 countries in Africa, Asia, USA, and Europe) to the Hajj [71] or returning from Hajj (Algeria, Chad, Comoros, Egypt, Ethiopia, Guinea, India, Indonesia, Libya, Mauritania, Nigeria, and Sudan) [70].

### 3.4. The Resistance Profile for AMR Bacteria

#### 3.4.1. Resistance Profile of Enteric Disease-Causing AMR Bacteria

Enteric bacteria have expressed beta-lactam resistance (including cephalosporin resistance) in 3050 out of 4220 isolates documented in 28 related studies reviewed [27,32,33,35,40,41,42,43,44,45,46,47,49,50,51,52,53,55,60,61,62,63,64,65,66]). In this review, most enteric bacteria were found in hospital settings, with a total of 3632 isolates within 18 studies [27,32,33,34,35,39,40,41,42,43,44,45,46,47,49,50,51,52]. Except for fosfomycin, resistance to antibiotics such as macrolides, lincosamides, and glycopeptides was reported only in hospital settings related to the Hajj; with 89, 67, and 158 isolates, respectively.

*Acinetobacter* spp. and *Pseudomonas aeruginosa* are examples of enteric organisms reported in the included studies. All AMR *Acinetobacter* spp. that are found related to the Hajj were 361 out of 526 *Acinetobacter* spp. isolates (68.63% [95% CI: 64.67%–72.60%]) reported in nine studies [27,32,39,42,45,50,59,63,64]. Most of the studies (five) were exploratory, as they tested for multiple possible organisms for isolates from multiple diseases and conditions [32,42,50,59,64]. Six studies found 348 out of 471 AMR *Acinetobacter* spp. (73.89% [95% CI: 69.92%–77.85%]) were isolated within hospital settings [27,32,39,45,50,63]. On the other hand, AMR *Pseudomonas aeruginosa* were a total of 466 out of 984 (47.36% [95% CI: 44.24%–50.48%]) *Pseudomonas aeruginosa* isolates. All were reported within six studies, and all were within hospital settings [32,35,42,43,44,51]. Most of the studies (13 out of 15 studies) that tested for AMR *Acinetobacter* spp. and *Pseudomonas aeruginosa* discussed the possible effect of overuse and misuse of antibiotics on the development of AMR enteric bacteria, and most emphasize the importance of surveillance and monitoring programs to reduce the burden of such resistance profiles.

#### 3.4.2. Resistance Profile of Respiratory Illness-Causing AMR Bacteria

There were 29 studies reporting AMR respiratory colonizing or infecting bacteria [21,22,27,28,30,32,36,37,38,42,43,48,52,54,55,57,61,63,64,65,67,68,69,70,71,72,73,74,75].

Methicillin-resistant *Staphylococcus aureus* (MRSA) and *Mycobacterium tuberculosis* (TB) are two important respiratory-related infections that merit further investigation.

Regarding MRSA, 14 out of 16 studies that tested for resistant *Staphylococcus aureus* found 933 out of 2164 MRSA isolates (43.11% [95% CI: 41.03%–45.20%]) within their studies [27,28,30,33,36,42,43,48,52,61,63,64,67,68]. Furthermore, nine studies reported 881 out of 1983 MRSA (44.43% [95% CI: 42.24%–46.61%]) isolated within hospital settings [27,28,30,35,36,42,43,48,52]. Table 3 compares the rate of selected AMR reported within the studies found in enteric and respiratory disease-causing bacteria isolated within hospital settings.

Regarding TB, there were only two studies included in the analysis that investigated resistance to TB medication [74,75]. These studies reported 110 out of 499 (22.04% [95% CI: 18.41%–25.68%]) TB isolates resistant to at least one TB medication (ethambutol, isoniazid, pyrazinamide, rifampicin, and/or streptomycin). Table 4 summarizes the available data on the AMR profile for TB.

## 4. Discussion

Our research provided an up-to-date account of the literature related to AMR bacteria acquisition and transmission associated with the Hajj pilgrimage. Enteric and respiratory disease-causing, beta-lactam resistance bacteria were the most reported organisms. In this section, we discuss preventive and surveillance measures that may be adopted to reduce the risk of AMR bacteria transmission during the Hajj.

### 4.1. Enteric Disease-Causing Beta-Lactam Resistance Bacteria

Our literature review found that 72.27% [95% CI: 70.92%–73.63%] of the tested enteric infection-causing bacteria were resistant to beta-lactams. This was similar to what other studies have found. For instance, Santos et al. (2020) found that phenotypically 74% and genetically 94% of tested enteric samples were resistant to at least one beta-lactam antibiotic [76]. Resistance to beta-lactams expressed by enteric bacteria poses health threats that increase morbidity and mortality rates [77,78], and a mismatch between treatment and susceptibility profile leads to a worse prognosis [77,78]. Studies showed that the mortality rate ranged from 42% to 100% for patients with enteric diseases who were infected with beta-lactam-resistant bacteria but were still treated with beta-lactam antibiotics [77,78]. We recommend that physicians considering using beta-lactams to treat pilgrims and their close contacts for enteric diseases consider the AMR risk profile of these patients, and factor this into their clinical decision-making accordingly. Implementation for such recommendation can be strengthened with the availability of data and enhanced surveillance of AMR for commonly used beta-lactams and communicating recommended protocols and guidelines between health authorities related to the Hajj and destination countries.

### 4.2. Respiratory Disease-Causing Beta-Lactam Resistance Bacteria

Our findings show that the most common (68.58% [95% CI: 66.87%–70.29%]) AMR expressed in respiratory infection-causing bacteria was beta-lactams and that this rate was significantly higher among cases detected in Makkah. The higher detected rate of beta-lactam resistance in Makkah compared to outside Makkah could be due to the high rate of respiratory infections among the Hajj pilgrims [79] or simply the result of measurement bias. The high rate of inappropriate antibiotic prescriptions for respiratory infections in the Hajj that is reported in literature [80,81], may also have contributed to this high detected rate of beta-lactam resistance within respiratory bacteria in the Hajj. Due to the high rate of respiratory infections reported in the Hajj [79], we recommend that health authorities in the Hajj support updating and developing guidelines, that are specific for the Hajj [82], for physicians when treating Hajj pilgrims with respiratory infections.

#### 4.2.1. AMR Isolated from Hospital Settings

We found 56.07% [95% CI: 55.06%–57.09%] of sampled bacteria collected in hospital settings in Makkah were resistant to at least one antibiotic; this is in line with the range reported elsewhere (27% to 82%) [83]. Moreover, our work found that the most common AMR bacteria was *Acinetobacter* spp. with about 88% of isolates collected resistant to at least one beta-lactam antibiotic. This is similar to a similar study’s findings [84]. Infection with *Acinetobacter* spp. is a serious health issue, as these organisms are robust and can survive in different environmental conditions, increasing the chance of antimicrobial resistance developing [85]. Therefore, hospitalized patients and pilgrims suspected to be infected with enteric bacteria, such as *Acinetobacter* spp. infections, are recommended to be tested for antimicrobial susceptibility to avoid inappropriate prescribing and thus minimise the risk of AMR development and improve treatment outcomes.

#### 4.2.2. Surveillance of AMR Bacteria

Sixty-five percent of the studies included in our review reported AMR detection through hospital-based research. Both routine and incidental hospital and community-based surveillance are crucial to assess, predict, and follow up on the development and transmission of AMR bacteria within the Hajj [86]. Moreover, tracking the use of antibiotics (e.g., prescription and dispensing) within the Hajj event may help find foci of unwanted antibiotic usage, such as self-medication and low regimen compliance in the Hajj [86,87]. To ensure adequate surveillance mechanisms are in place to detect AMR associated with the Hajj a comprehensive strategy integrated with broader health protection planning measures are essential. A comprehensive surveillance strategy should incorporate routine hospital-based data collection, community-based surveillance (i.e., private health providers and laboratories), and active case finding when required.

#### 4.2.3. Preventive Measures for AMR Bacteria

The prevalence of AMR transmission at the Hajj should be emphasised on prevention, early detection, and rapid response. Measures that should be considered include the development of policies that require mandatory vaccination (e.g., meningococcal) and prophylaxis before coming to Hajj; strategies to strengthen hospitals’ infection controls; the development of Hajj-specific guidelines for AMR prevention, detection and control; enhancement of hospital- and community-based surveillance for AMR; streamlining testing processes; establishment of governance arrangements that support the adoption of novel (or improved) methods of tackling AMR risk; and investing in social, clinical, and operational research to better understand transmission dynamics.

### 4.3. Limitations

This study has several limitations. First, most of the included studies provided limited information about participants’ demographics. Demographic data (such as age and gender) are important, as some diseases have high occurrence rates associated with specific ages. Second, we relied on the information provided in the reviewed articles and could not interrogate or validate their findings. Third, some studies were excluded as they were not available in English, which may have resulted in relevant information being missed. Forth, most studies were based on data collected as part of the research conducted in Makkah and hence, AMR detection associated with Makkah is likely overrepresented. Finally, presence of bias was not universally discussed in the manuscripts review. Existence of bias in the literature reviewed may impact the validity and generalizability of our results.

## 5. Conclusions

This scoping review provides a point-in-time collation of available scientific information related to AMR transmission associated with the Hajj. It provides an accessible resource for researchers and practitioners seeking insights into AMR-associated incidence and burden and identifies knowledge gaps that may be filled. Finally, the work offers generalizable advice that those responsible for the management of AMR at mass gatherings may refer to inform their efforts.

## Figures and Tables

**Table 2:** The number of antimicrobial resistant (AMR) isolates that were reported in literature, published from 1990 to 2021, related to AMR bacteria within the Hajj. Each bacterial species isolates are categorized by their resistance to the five main antibiotic classes.

Organism(s) Name	Aminoglycoside		Beta-Lactams		Macrolides		Quinolones		Sulphonamides	
	Number of Reported AMR Isolates	Reported in (*n*) Studies	Number of Reported AMR Isolates	Reported in (*n*) Studies	Number of Reported AMR Isolates	Reported in (*n*) Studies	Number of Reported AMR Isolates	Reported in (*n*) Studies	Number of Reported AMR Isolates	Reported in (*n*) Studies
Enteric bacteria										
*Acinetobacter* spp.	393	8	491	9	0	0	316	7	323	5
*Bacillus* spp.	0	0	3	1	1	1	0	0	1	1
*Bacteroides* spp.	3	1	2	1	0	0	1	1	1	1
*Brachybacterium* spp.	0	0	1	1	0	0	0	0	1	1
*Burkholderia* spp.	2	1	2	1	0	0	1	1	1	1
*Citrobacter* spp.	4	2	8	2	0	0	3	1	2	1
*Enterobacter* spp.	25	4	69	6	0	0	18	3	32	3
*Enterococcus* spp.	68	2	176	2	56	2	39	1	21	1
*Escherichia coli*	489	9	726	13	18	1	407	9	426	5
*Klebsiella* spp.	339	5	475	8	0	0	264	6	242	4
*Proteus* spp.	51	2	74	4	0	0	45	2	29	2
*Pseudomonas* spp.	736	5	800	5	0	0	397	4	240	2
*Salmonella* spp.	3	2	34	3	0	0	0	0	7	1
*Serratia* spp.	4	1	21	1	0	0	1	1	7	1
*Shigella* spp.	0	0	9	1	0	0	0	0	0	0
*Vibrio cholerae*	0	0	3	1	0	0	0	0	3	1
*Yersinia enterocolitica*	0	0	3	1	0	0	0	0	0	0
Respiratory bacteria										
*Haemophilus influenzae*	38	2	47	2	0	0	15	1	38	2
*Neisseria meningitidis*	0	0	5	2	0	0	3	1	45	1
*Staphylococcus* spp.	621	9	1748	16	818	10	57	4	696	6
*Stenotrophomonas maltophilia*	1	100	1	100	1	100	1	100	1	100
*Streptococcus* spp.	113	1	146	1	266	1	43	1	380	1
Other bacteria										
*Brucella* spp.	1	1	1	1	0	0	0	0	1	1
*Ewingella americana*	1	1	1	1	0	0	0	0	1	1
*Helicobacter pylori*	0	0	2	1	0	0	0	0	2	1
*Microbacterium* spp.	0	0	6	1	1	1	0	0	3	1
*Micrococcus* spp.	0	0	1	1	0	0	0	0	0	0
*Shewanella xiamenensis*	1	1	1	1	0	0	0	0	1	1

NA = not applicable; spp. = species; *n* = number of studies.

**Table 3:** Rates of selected antibiotic resistances for enteric and respiratory disease-causing bacteria isolated in hospital settings.

	AMR Rate among Respiratory Disease–Causing Bacteria Isolated In Hospital Settings (*n* = 1824)			AMR Rate among Enteric Disease–Causing Bacteria Isolated in Hospital Settings (*n* = 3632)		
Antibiotic Class	*n*	(%)	95% CI (%)	*n*	(%)	95% CI (%)
Aminoglycoside	561	30.76	[28.64–32.87]	1996	54.96	[53.34–56.57]
Beta–lactams	1424	78.07	[76.17–79.97]	2730	75.17	[73.76–76.57]
Lincosamides	359	19.68	[17.86–21.51]	67	1.84	[1.41–2.28]
Macrolides	693	37.99	[35.77–40.22]	89	2.45	[1.95–2.95]
Quinolones	22	1.21	[0.71–1.71]	1415	38.96	[37.37–40.55]
Sulphonamides	767	42.05	[39.79–44.32]	1291	35.55	[33.99–37.10]
Tetracyclines	237	12.99	[11.45–1454]	486	13.38	[12.27–14.49]

**Table 4:** Resistance rates against anti-Mycobacterium Tuberculosis drugs for Mycobacterium Tuberculosis.

	Resistance in TB Isolates		
	*n*	Rate (%)	95% CI (%)
AMR to anti-TB drug			
Ethambutol	33	6.61	[4.43–8.79]
Isoniazid	30	6.01	[3.93–8.10]
pyrazinamide	25	5.01	[3.10–6.92]
Rifampicin	25	5.01	[3.10–6.92]
Streptomycin	46	9.22	[6.68–11.76]
Number of anti-TB drugs with AMR			
Monoresistance (one drug only)	71	14.23	[11.16–17.29]
Two drugs only	25	5.01	[3.10–6.92]
Three or more	14	2.81	[1.36–4.25]

AMR = antimicrobial resistance; TB = *Mycobacterium Tuberculosis.*

## Data Availability

Not applicable.

## Supplementary Materials

The following supporting information can be downloaded at: https://www.mdpi.com/article/10.3390/ijerph192114134/s1, S1: Search Terms; S2: Extracted data. S3: PRISMA checklist.
