# Supplementary material for: Transmission of Antimicrobial Resistant Bacteria at the Hajj: A Scoping Review
Source: Int J Environ Res Public Health. 2022 Oct 29;19(21):14134. doi: 10.3390/ijerph192114134 (PMC9658569; doi:10.3390/ijerph192114134)
Supplement: Supplementary file 1 [file ijerph-19-14134-s001.zip › S1.pdf]

Search Terms:

1. **MEDLINE - primary component of PubMed**

("arafat"[All Fields] OR "hadj*"[All Fields] OR "haj"[All Fields] OR "hajj*"[All Fields] OR "makkah"[All Fields] OR "mecca"[All Fields] OR "mina"[All Fields] OR "muzdalefah"[All Fields] OR "muzdalifah"[All Fields] OR "pilgrim*"[All Fields]) AND ("appl magn reson"[Journal] OR "altern med rev"[Journal] OR "amr"[All Fields] OR ("anti bacterial agents"[Pharmacological Action] OR "anti bacterial agents"[MeSH Terms] OR ("anti bacterial"[All Fields] AND "agents"[All Fields]) OR "anti bacterial agents"[All Fields] OR "antibacterial"[All Fields] OR "antibacterials"[All Fields] OR "antibacterially"[All Fields]) OR "antibiotic*"[All Fields] OR "antimicrob*"[All Fields] OR ("communicable diseases"[MeSH Terms] OR ("communicable"[All Fields] AND "diseases"[All Fields]) OR "communicable diseases"[All Fields] OR ("communicable"[All Fields] AND "disease"[All Fields]) OR "communicable disease"[All Fields]) OR "multidrug*"[All Fields] OR "multi drug*"[All Fields]) AND ("inspect*"[All Fields] OR "monitor*"[All Fields] OR "observ*"[All Fields] OR "surve*"[All Fields]) AND ("resistan*"[All Fields] OR "sensitiv*"[All Fields] OR "suscept*"[All Fields])

2. **EMBASE - Ovid**

#	**Searches**	**Results Type**
1.	arafat.mp.	34
2.	hadj*.mp.	269
3.	haj.mp.	134
4.	hajj*.mp.	1047
5.	makkah.mp.	629
6.	mecca.mp.	412
7.	mina.mp.	585
8.	muzdalefah.mp.	1
9.	muzdalifah.mp.	1
10.	pilgrim*.mp.	1588
11.	**1 or 2 or 3 or 4 or 5 or 6 or 7 or 8 or 9 or 10**	3575
12.	AMR.mp.	9613
13.	antibiotic*.mp.	903210
14.	antimicrob*.mp.	290718
15.	communicable disease.mp. or communicable disease/	49388
16.	multidrug*.mp.	126921

17.	multi-drug*.mp.	20315
18.	antibacterial.mp. or antiinfective agent/	338834
19.	**12 or 13 or 14 or 15 or 16 or 17 or 18**	1324501
20.	resistan*.mp.	1748774
21.	sensitiv*.mp.	2388327
22.	suscept*.mp.	692185
23.	**20 or 21 or 22**	4246427
24.	inspect*.mp.	73358
25.	monitor*.mp.	1532082
26.	observ*.mp.	5310490
27.	surve*.mp.	1952735
28.	**24 or 25 or 26 or 27**	8256890
29.	**11 and 19 and 23 and 28**	58

3. CINAHL – EBSCO

#	Query	Results
S5	S1 AND S2 AND S3 AND S4	34
S4	inspect* OR monitor* OR observ* OR surve*	1,081,837
S3	resistan* OR sensitiv* OR suscept*	443,228
S2	AMR OR antibacterial OR antibiotic* OR antimicrob* OR communicable disease OR multidrug* OR multi-drug*	131,273
S1	arafat OR hadj* OR haj OR hajj* OR makkah OR mecca OR mina OR muzdalefah OR muzdalifah OR pilgrim*	10,167

4. Scopus – Elsevier

((TITLE-ABS-KEY (arafat) OR TITLE-ABS-KEY (hadj*) OR TITLE-ABS-KEY (haj) OR TITLE-ABS-KEY (hajj*) OR TITLE-ABS-KEY (makkah) OR TITLE-ABS-KEY (mecca) OR TITLE-ABS-KEY (mina) OR TITLE-ABS-KEY (muzdalefah) OR TITLE-ABS-KEY (muzdalifah) OR TITLE-ABS-KEY (pilgrim*))) AND ((TITLE-ABS-KEY (amr) OR TITLE-ABS-KEY (antibacterial) OR TITLE-ABS-KEY (antibiotic*) OR TITLE-ABS-KEY (antimicrob*) OR TITLE-ABS-KEY (communicable AND disease) OR TITLE-ABS-KEY (multidrug*) OR TITLE-ABS-KEY (multi-drug*))) AND ((TITLE-ABS-KEY (resistan*) OR TITLE-ABS-KEY (sensitiv*) OR TITLE-ABS-KEY (suscept*))) AND ((TITLE-ABS-KEY (inspect*) OR TITLE-ABS-KEY (monitor*) OR TITLE-ABS-KEY (observ*) OR TITLE-ABS-KEY (surve*)))
